# Root ZX Electronic Foramen Locator: An Ex Vivo Study of Its Three Models' Precision and Reproducibility

**DOI:** 10.1155/2017/5893790

**Published:** 2017-03-07

**Authors:** Bernardo Almeida Aguiar, Rafael Santos Reinaldo, Luciana Maria Arcanjo Frota, Mônica Sampaio do Vale, Bruno Carvalho de Vasconcelos

**Affiliations:** ^1^Postgraduate Program in Dentistry, Federal University of Ceará, Fortaleza, CE, Brazil; ^2^Private Practice, Fortaleza, CE, Brazil; ^3^School of Pharmacy, Dentistry and Nursing, Federal University of Ceará, Campus Fortaleza, Fortaleza, CE, Brazil; ^4^School of Dentistry of Sobral, Federal University of Ceará, Campus Sobral, Sobral, CE, Brazil

## Abstract

Although Root ZX is considered the gold standard electronic foramen locator (EFL), two variations of this device were launched, however without different operating mechanisms. This investigation aims to evaluate the precision of Root ZX (RZX), Root ZX II (RII), and Root ZX Mini (RM) EFLs. After access cavity preparation, 32 mandibular single rooted human premolars had their real length measured with the aid of a #15 K-type manual file under magnification (25x). Electronic measurements were performed by the devices in an alternate order until the apical foramen was reached (0.0). Each measurement was performed with adjusted file to the real length of the teeth and verified with a digital caliper. The accuracy of the EFLs was 68.8% (RZX), 65.8% (RII), and 68.8% (RM), considering ±0.5 mm as a margin of tolerance. The mean errors of the devices were 0.37 ± 0.25 mm (RZX), 0.41 ± 0.34 mm (RII), and 0.32 ± 0.28 mm (RM). ANOVA and Tukey test were applied to analyze the obtained data, which showed that there were no statistically significant differences among the locators (*P* > .05). It can be concluded that the three tested devices demonstrated precise measurements of the real length of the canal without performance differences among them.

## 1. Introduction

The success of the endodontic treatment depends on the execution of a series of linked steps. Among them, the determination of the real root canal length is essential to establish the limits of the chemical-mechanical disinfection and of the obturation, which are key points to avoid injuries to the periapical tissues [1–3].

The apical constriction is the shortest diameter of the root canal, where the transition from the pulp to the periodontal tissue is found [1]. This anatomic landmark located 0.5 mm to 1.0 mm from the apical foramen is considered the ideal instrumentation and obturation limit for the root canal therapy [1]. The use of electronic devices to determine the root canal length was firstly proposed by Custer [4] in 1918; however, just after Suzuki's [5] investigations of the electrical resistance properties of the oral tissues, the first electronic foramen locator (EFL) was launched. The first and second operation mechanisms developed by the measurement devices were based on resistance and impedance principles. The main deficiency of both mechanisms was the imprecision in the presence of electrolytes, which was exceeded by the arising of a new method. The EFL Root ZX (J. Morita Corp., Tokyo, Japan), created by Kobayashi and Suda [6], introduced the frequency-based impedance method; this device operates an impedance ratio method calculating the ratio between the impedances measured in two different frequencies (8 KHz and 0.4 KHz) [7]. The Root ZX has received considerable attention from the scientific community since its introduction because this device shows excellent performance, which makes it the gold standard EFL [8–10].

The J. Morita Corporation discontinued the production of the original Root ZX launching two different devices, Root ZX II and Root ZX Mini. They were created based on the same functioning method of the Root ZX, however, this time with the possibility of attaching the EFL to a motor for mechanical instrumentation (Root ZX II) and with the advantage of being a miniaturized version (Root ZX Mini) [9, 10]. Currently, the precision and reproducibility of the measurements made by these three models of Root ZX were not compared in a single study.

Therefore, considering the great popularity of the Root ZX models with the clinicians and endodontists and also considering the importance of the correct determination of the endodontic root canal real length, the aim of this investigation is to evaluate the precision of three EFLs, Root ZX, Root ZX II, and Root ZX Mini, comparing their accuracy to locate the apical foramen.

## 2. Materials and Methods

Thirty-two mandibular single rooted human premolar teeth with completed formed apices that were indicated for extraction due to orthodontic reasons were selected to compose the sample of this study. All the teeth were healthy and correspond to Vertucci type I; they also do not present accentuated dilacerations (<30°).

To remove the residual tissues adhered to the roots, the specimens were immersed in 2.5% sodium hypochlorite solution for 4 hours. When present, dental calculus and other residues were removed with ultrasonic tips. Subsequently, the access cavity preparation was performed with the use of diamond points (#1012, #3081; KG Sorensen, Cotia, SP, Brazil) under constant irrigation. Flat surfaces were created on the occlusal surface of the teeth to serve as anatomical references. The canals were initially explored with a #10 K-type manual file (Dentsply Maillefer, Ballaigues, Switzerland). The remains of the pulp tissue were gently removed. The teeth were numbered and had their real canal length determined by inserting a file into the root canal until its tip could be seen reaching the apical foramen under the 25x magnification of a clinical microscope (DF Vasconcellos, São Paulo, SP, Brazil). For this procedure, the teeth were adapted to a special support allowing good and standardized observation of the apical foramens. These measurements were performed by an experienced end previous calibrated endodontist. The distance between the tip and the rubber stop was determined using a digital caliper with ±0.001 mm precision (FNCL, Worker Gage, Esteio, Brazil); this length was registered as the real length of the root canals.

The instrumentation of the cervical and medium thirds of the canals was performed using Protaper SX instruments (Dentsply Maillefer), which were inserted until reaching 5 mm far from the real length of the canals. The 2.5% sodium hypochlorite solution (Asfer Indústria Química Ltda., São Caetano do Sul, SP, Brazil) was employed as an irrigant.

The apexes of the roots and a lip clip were introduced into freshly manipulated alginate (<30 min) (Jeltrate II; Dentsply, Petrópolis, RJ, Brazil) in order to allow electronic measurements. After the irrigation of 0.5 mL of the irrigant, the measurements were performed with endodontic hand files adapted to the diameter of the apical third of the canals. For each group of 5 specimens, the measurements were performed in triplicate, alternating the use of the different models of Root ZX EFL. The measurements were determined after 5 seconds of stability of the device; after that, the file was removed from the canal and its length verified with a digital caliper.

The mean values of the devices measurements were calculated using Excel 2007 (Microsoft Corp., Redmond, WA) and compared to the real length of the canals. The analysis of these calculations allowed the determination of the precision of each device regarding their discrepancies in mm. Considering the parametric nature of the data, they were submitted to ANOVA and to Tukey's test with significance level of *P* < .05.

## 3. Results

The precision of the Root ZX models can be observed in Table 1 which presents the mean, the standard deviation, and the error range found in the electronic measurements (absolute values of the discrepancies). There were no statistical differences between the measurements obtained by Root ZX, Root ZX II, and Root ZX Mini (*P* > .05).

Table 2 lists the distribution of the measurements obtained by the use of the foramen locators. The percentages of precision of the devices were 68.8% and 100% (Root ZX), 65.8% and 96.9% (Root ZX II), and 68.8% and 100% (Root ZX Mini), considering ±0.5 mm and ±1.0 mm as the error range, respectively. Measurements in which the endodontic file went out of the apex foramen were observed in 3.1% of the procedures with Root ZX, 12.5% for the ones with Root ZX II, and 9.6% for the Root ZX Mini.

## 4. Discussion

This investigation evaluated the different models of Root ZX ELF correlating the results obtained with the precision of the devices in determining the root length at the foraminal level (0.0). To the best of our knowledge, there is no study in the literature comparing these three Root ZX locator models in a single evaluation. The reproduction of the alginate model [11], using cervical preflaring [12], performing the measurements with endodontic files introduced into the canal until the apical foramen [13, 14] was used to guarantee the reproducibility of the experimental protocol, as well as permit results extrapolation for clinical conditions [15].

Considering the real root canal length measurement importance, here an experienced endodontist with the aid of a clinical microscope performed it carefully. The measurement method presents huge importance since the EFLs determinations errors will be based on it. A great number of studies used the visual method for its evaluations [13, 16–19]. Piasecki et al. (2016), among other evaluations, performed a comparison of measurement methods, including the visual one, stating that any significant difference could be found between them, thus validating the results of the present study [18].

Nowadays, the EFLs are indispensable tools to perform the odontometry step during the endodontic treatment [1, 19]. Even though the different currently available EFLs present different precision rates, they are considered safe, trustable, and precise to be used. Each manufacturer suggests that their device was better than the other ones; these EFLs operate several mathematical interpretations of the impedance measured justifying little precision differences. Even devices that operate similar mechanisms could present quite differences as Propex II and RomiApex A-15 [13], thus highlighting the relevance of the present study.

Precision of 68.8% and 65.7% was offered by the Root ZX Mini and Root ZX II EFLs, respectively, considering ±0.5 mm as error range. This precision along with the mean error near 0.0 could be attributed to the devices mechanism. These devices operate a method similar to the one performed by the Root ZX, which exhibited in this study a rate of 68.8% of precision. These results corroborate previously published investigations that highlight the Root ZX quality [8, 10, 15]. da Silva and Alves (2014) [20], who also used an error range of ±0.5 mm, obtained precision of 62.5% for the Root ZX II, 56.2% for the Root ZX Mini, and 50% for the RomiApex A-15.

Nekoofar et al. (2006) stated that the precision of the EFLs varies when different protocols of use are adopted [1]. The results of our investigation show this variability when two error ranges are considered (±0.5 mm and ±1.0 mm), which generates precision values of 68.8% and 100% for the Root ZX, 65.8% and 96.9% for the Root ZX II, and 68.8% and 100% for the Root ZX Mini, respectively, to the error ranges. These variations were also reported by Pascon et al. (2009) [7], who found higher precision discrepancies when considering different error ranges, since these authors obtained precision rates of 39% and 90% for the locator Root ZX II, 31% and 82% for the Raypex 5, and 37% and 73% for the Elements Diagnostic Unit and Apex Locator.

Measurements in which the endodontic file surpassed the apical foramen were found in 3.1% (Root ZX), 12.5% (Root ZX II), and 9.6% (Root ZX Mini). These findings are in accordance with the results obtained by D'Assunção et al. (2007) [21], ElAyouti et al. (2005) [22], and Pascon et al. (2009) [7] who reported a percentage of 2.6% to 30% of measurements in which the file reached the periapical tissues. These results are important since they represent lower chances of performing over instrumentations, mainly for those clinicians that rely only on the use of EFLs to perform the odontometry [13, 23].

Comparing the precision of EFLs is a challenge once a wide range of variables can affect the measurements of the root canal length. The results obtained by the use of the Root ZX were similar to the ones found in other previously published studies [24, 25]. As for its successors, both the Root ZX II, previously evaluated in other studies [14, 19], and the Root ZX Mini [19, 23] offered results similar to those offered by the original Root ZX. Up to now, no study has related the accuracy of the three Root ZX models; this way, the results indicate that although they present differences regarding the electronic components with which they are constituted, since they offer different variations of use and/or characteristics, the two models currently available offer similar determination and reliability standards to the original equipment. These findings reinforce the safety of the clinical use of these devices.

## 5. Conclusion

Under the conditions of the present study, it can be concluded that the three models of the Root ZX EFL demonstrated similar and adequate precision when performing root canal length measurement at the apical foramen level.

## Figures and Tables

**Table 1:** Mean, standard deviation, and error range of the experimental groups.

	*n*	Mean	Standard deviation	Minimum	Maximum
Root ZX	32	0.37^a^	0.25	0.00	0.85
Root ZX II	32	0.41^a^	0.34	0.00	1.15
Root ZX Mini	32	0.32^a^	0.28	0.00	0.85

Different superscript letters indicate the presence of a statistically significant difference among the experimental groups (*P* < .05).

**Table 2:** Position of the tip of the endodontic file relative to the apex foramen in the measurements performed until 0.0.

Position	Root ZX		Root ZX II		Root ZX Mini	
	*n*	%	*n*	%	*N*	%
<−1.0^*∗*^	0	0.0	0	0.0	0	0.0
−1.0 to −0.51^*∗*^	9	28.1	9	28.1	8	25.5
−0.5 to −0.01^*∗*^	16	50.0	12	37.5	12	37.5
0	6	18.8	7	21.9	8	25.0
0.01 to 0.05	0	0.0	2	6.3	2	6.3
0.51 to 1.0	1	3.1	1	3.1	2	6.3
1.0	0	0.0	1	3.1	0	0.0

^*∗*^Negative values indicate that the endodontic file is positioned inside the root canal.
